# Defying the economic odds: vocational recovery as a clinical imperative in severe mental disorders

**DOI:** 10.3389/fpubh.2026.1805221

**Published:** 2026-04-24

**Authors:** Haewon Byeon

**Affiliations:** Worker's Care & Digital Health Lab, Department of Future Technology, Korea University of Technology and Education (KOREA TECH), Cheonan, Republic of Korea

**Keywords:** individual placement and support, severe mental disorders, social determinants, unemployment, vocational recovery

## Introduction

For individuals living with severe mental disorders (SMDs) such as schizophrenia, exclusion from the labor market creates a secondary pathology: the erosion of identity, autonomy, and social purpose. Although many people with SMDs want to work, competitive employment remains difficult to attain. The Individual Placement and Support (IPS) model has challenged the assumption that illness severity alone explains poor labor-market participation in the United States and Northern Europe (1, 2). However, doubts about IPS often persist in Southern Europe, where structural unemployment has been high and conventional pre-employment approaches have remained influential (1, 4).

The recent randomized controlled trial by Rodríguez Pulido et al. (3) confronts this skepticism directly. Conducted in Tenerife, Canary Islands, the trial examined adults with SMDs recruited from public Community Mental Health Teams and basic health areas in a high-unemployment island setting (3). This commentary focuses specifically on the implications of that Canarian trial for vocational recovery in adults with SMDs; it is not intended as a general review of all vocational rehabilitation systems in Spain or Southern Europe.

The Canary Islands context deserves closer attention because it differs in important ways from how IPS is often discussed in the United States and other settings. In Tenerife, IPS was first implemented in 2004 and has since been coordinated by the Canarian Health Service, together with SINPROMI and the University of La Laguna, with stable public funding and integration into mental health services (3). This means the Tenerife study reflects not simply a difficult labor market, but a territorially organized public partnership within a Spanish autonomous community. That distinction matters. In Spain, supported employment and IPS implementation have developed unevenly across regions and often alongside more traditional train-then-place models (4, 5). In Catalonia, for example, IPS implementation between 2013 and 2017 required explicit coordination across the health, labor, and social service sectors to move services beyond conventional pre-employment pathways (4). The Canary Islands also have a longer regional history of supported employment through SINPROMI, which has delivered employment–training support and job-coach assistance in Tenerife since the 1990s (5). Moreover, Tenerife is not a *de novo* IPS site: earlier studies from the island had already reported favorable supported-employment outcomes for people with severe mental disorders (6).

## The myth of the “ready” patient and the “healthy” economy

Traditional vocational rehabilitation (VR) follows a stepwise logic: stabilize symptoms, train in a sheltered environment, and then search for employment. This “train-then-place” approach assumes that patients need protection from the stresses of the open market (7). Rodríguez Pulido et al. (3) provide data that dismantles this protective logic. Their control group, receiving standard VR, achieved a competitive employment rate of only 15.8%. In stark contrast, the IPS group achieved 75% (3).

Importantly, the comparison condition in Tenerife was not the absence of service, but a conventional vocational rehabilitation pathway that more closely resembles the preparatory logic still found in many non-IPS systems (4, 7). This disparity highlights a critical failure in traditional public health approaches to disability. By waiting for patients to be “ready” or for the economy to be “receptive,” we inadvertently enforce chronicity. The success of the IPS arm in Tenerife suggests that the active ingredient is not the number of available jobs in the community, but rather the integration of employment specialists into clinical teams. These specialists navigate the labor market with the client, bypassing bureaucratic friction that often defeats individuals with cognitive or motivational deficits. The study proves that high regional unemployment is a variable, not a veto.

## Employment as a clinical intervention

Perhaps the most compelling aspect of the Tenerife trial is not the employment rate itself, but the secondary health outcomes. A pervasive fear among clinicians is that the pressure of competitive employment will trigger psychotic relapses (8). The data from the Rodríguez Pulido trial suggest the opposite (3).

Participants in the IPS group did not experience higher rates of adverse events or hospitalizations. Instead, they demonstrated significant improvements on the Global Assessment of Functioning (GAF) compared to the control group. Furthermore, those who obtained employment—regardless of their group assignment—reported greater gains in quality of life (3). This reinforces the concept of “work as medicine.” The structure, social contact, and financial autonomy provided by a job act as stabilizers (9). Unemployment, conversely, appears to be toxic exposure. By denying access to rapid job placement, health systems may be withholding a potent therapeutic tool that addresses the negative symptoms of schizophrenia, often resistant to pharmacotherapy.

## Limitations and the need for long-term data

While the results are promising, we must approach them from a measured perspective. The study's sample size (*n* = 63) is modest, and the follow-up period is limited to 6 months. In the realm of vocational recovery, getting a job is only the first hurdle; keeping it is the marathon. The “tenure” of employment for people with SMDs is notoriously fragile (10). Without data extending to 12 or 24 months, we cannot fully assess whether the IPS placements in this high-unemployment setting are sustainable or if they represent short-term successes in a volatile market.

Additionally, the dropout rate of approximately 32% demands scrutiny. While the authors note that attrition was not associated with baseline clinical characteristics, it raises questions about engagement. Does the rapid pace of IPS overwhelm a subset of patients? Or does the logistical burden of participating in such programs deter those with fewer resources? Future research must look beyond the “completers” to understand who the model fails to reach and why.

A further limitation concerns contextual generalizability. Tenerife appears to benefit from unusually strong coordination among public mental health teams, employment specialists, and regional disability-support institutions (3–5). That organizational architecture may partly explain the favorable results and may not be present in the same form in other Spanish regions or other countries. For that reason, the Canary Islands are best understood here as an instructive case rather than a simple proxy for all high-unemployment settings.

## Policy implications: action over hesitation

The findings by Rodríguez Pulido et al. (3) carry immediate weight for health policy in regions facing economic stagnation. Policymakers often delay implementing IPS, arguing that it is a luxury in full-employment economies. This study suggests that such hesitation is scientifically unfounded.

At the same time, the policy lesson should be framed carefully. The Tenerife findings are best read as evidence that IPS can succeed in a high-unemployment island region when it is embedded in a mature, well-coordinated public support system (3–6). They do not mean that all vocational rehabilitation systems in Spain, Europe, or the United States are institutionally equivalent. If we accept that social determinants are primary drivers of health, then facilitating access to work is as crucial as ensuring access to medication. The Canarian experience suggests that clinical recovery and vocational inclusion can proceed in parallel even under adverse economic conditions, but outcomes are likely to depend on local governance, cross-sector coordination, and fidelity to the IPS model (3–6). Public health systems should prioritize funding for integrated employment services, recognizing that the cost of inaction—measured in lost years of healthy life and social dependence—far outweighs the investment in support. We must stop protecting patients from the “risks” of employment and start protecting them from the certainty of decline associated with exclusion.

In summary, the Tenerife trial strengthens the case for treating employment as a clinical and public-health priority for adults with SMDs, while also underscoring that the implementation context matters. The contribution of this commentary is therefore not to generalize beyond the evidence, but to show why the Canary Islands offer a particularly important and policy-relevant test case for vocational recovery in severe mental disorders.
